# A Convolutional Neural Network for Automatic Tooth Numbering in Panoramic Images

**DOI:** 10.1155/2021/3625386

**Published:** 2021-12-14

**Authors:** María Prados-Privado, Javier García Villalón, Antonio Blázquez Torres, Carlos Hugo Martínez-Martínez, Carlos Ivorra

**Affiliations:** ^1^Asisa Dental, Research Department, C/José Abascal, 32, 28003 Madrid, Spain; ^2^Department of Signal Theory and Communications, Higher Polytechnic School, Universidad de Alcalá de Henares, Ctra. Madrid-Barcelona, Km. 33, 600, 28805 Alcalá de Henares, Spain; ^3^Department of Continuum Mechanics and Structural Analysis, Higher Polytechnic School, Carlos III University, Avenida de la Universidad 30, Leganés, 28911 Madrid, Spain; ^4^SysOnline, Murcia, Spain; ^5^Faculty of Medicine, Universidad Complutense de Madrid, Plaza de Ramón y Cajal, S/N, 28040 Madrid, Spain

## Abstract

Analysis of dental radiographs and images is an important and common part of the diagnostic process in daily clinical practice. During the diagnostic process, the dentist must interpret, among others, tooth numbering. This study is aimed at proposing a convolutional neural network (CNN) that performs this task automatically for panoramic radiographs. A total of 8,000 panoramic images were categorized by two experts with more than three years of experience in general dentistry. The neural network consists of two main layers: object detection and classification, which is the support of the previous one and a transfer learning to improve computing time and precision. A Matterport Mask RCNN was employed in the object detection. A ResNet101 was employed in the classification layer. The neural model achieved a total loss of 6.17% (accuracy of 93.83%). The architecture of the model achieved an accuracy of 99.24% in tooth detection and 93.83% in numbering teeth with different oral health conditions.

## 1. Introduction

Modern dentistry employs computer-assisted procedures in common dental treatments such as surgical planning, postoperative assessment, mechanized dental implants, and orthodontic planning [1].

The numbering of teeth in dental radiology is a routine evaluation that takes up time. Nowadays, dental images have been used combined with artificial intelligence in many applications such as dental diagnosis and dental treatment [2, 3]. Numbering teeth is required, for example, to identify human dental images, in routine dental procedures, maxillofacial surgical applications, and teeth generic modelling [4].

A large number of studies have been developed employing deep learning to reduce the workload of professionals and to recognize certain features [5]. Neural networks used for image recognition have evolved over time: initially started using Regions with Convolutional Neural Networks (R-CNNs) for classification tasks and continued with the use of fast R-CNN for classification and detection [6, 7]. Presently, deep learning methods based on convolutional neural networks are being widely used in the field of medical image analysis [8]. This study is employed to detect and number teeth in panoramic images.

The objective of this study was to modify the neural network used in a previous study by the authors [9], which obtained a precision of 99.24% in detecting the presence or absence of a tooth, to be used in the numbering of teeth in a panoramic image according to the Federation Dentaire Internationale (FDI) teeth numbering system.

## 2. Materials and Methods

### 2.1. Study Design

This study used a dataset of anonymized and categorized panoramic dental images. A CNN was first constructed to detect the presence or absence of teeth on the radiography and was later modified to number teeth according to FDI classification. Reporting of this study follows the STARD guideline [10].

### 2.2. Image Dataset

Panoramic images were taken from Asisa Dental S.A.U. centers in the Community of Madrid (Spain). These images are completely anonymized by CareStream Health Spain SA (Pozuelo de Alarcón, Madrid, Spain). No additional information such as name, gender, age, or when the image was taken appears in the database. Data collection was ethically approved (Ethics Committee of Research with Regional Medicines of the Community of Madrid (CEIm-R)) on June 15, 2018. The requirement to obtain informed consent from patients was waived by the ethics committee.

The inclusion criteria of the image database employed in the present study were adults older than 18 years. The exclusion criteria of images were edentulous patients, those images with temporary teeth and poor definition, images with removable prostheses, or images with only the presence of implants, computerized axial tomography (CAT), and radiography with overlap or objects out of the imaging plane.

Each image was revised by two examiners with more than three years of experience in general dentistry. The examiners evaluated the image database through a visualization program created to collect the information. The inclusion criteria were panoramic images from adults older than 18. The exclusion criteria were images of edentulous patients, those with temporary teeth, poor definition, with removable prostheses, or with only presence of implants. Computerized axial tomographies (CAT) were also excluded. Radiographies with overlap or objects out of the imaging plane were excluded.

**Figure 1:**
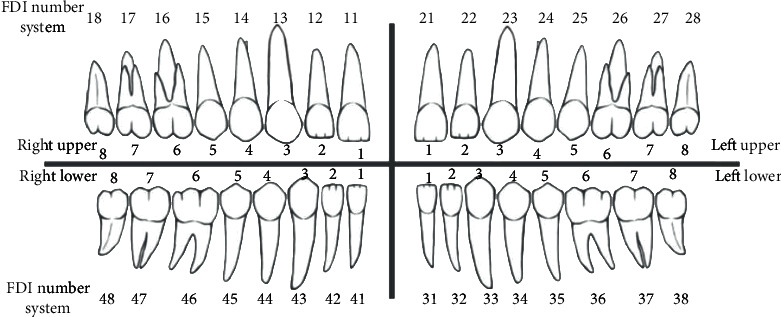
FDI classification system: Q1: 11–18 = right upper 1–8, Q2: 21–28 = left upper 1–8, Q3: 31–38 = left lower 1–8, Q4: 41–48 = right lower 1–8; 1. Central incisor. 2. Lateral incisor. 3. Canine. 4. First premolar. 5. Second premolar. 6. First molar. 7. Second molar. 8. Third molar.

For this study, the 5,121 8-bits images employed in a previous published manuscript by the authors [9] were used to start the image database in the present study. A set of 2,230 correctly demarcated samples was obtained. Of these 2,230 samples, those with 28 or more teeth were selected. It was possible to identify 1,617 samples with these characteristics, from which those that had metallic parts were filtered, of which 1,217 samples suitable for training and validation of the final FDI detection and assignment model were obtained (Figure 1).

The number of existing teeth in the 2,230 images, distributed by their FDI, is detailed in Table 1. As can be seen, for all quadrants, the number of pieces 1 to 7 is quite homogeneous. However, in the case of piece 8, it is not always categorized by the experts, and there are also fewer cases.

### 2.3. CNN Architecture

The categorized panoramic radiographs are used as an input for the neural network architecture presented. The system outputs the bounding boxes and the teeth number for all detected teeth on the image.

The algorithms were running backend on TensorFlow version 1.14 and Tensorflow 2.2., and the operating system was Windows 10 and Ubuntu 18.4. In the final step, it was tested in the cloud (AWS) on instance p3.8large (4 GPU's Tesla V100, 64 GB GPU memory, instance memory: 244 GB, vCores of instance: 32), with the deep learning AMI using the virtual environment of conda tensorflow_p36.

The neural network consists of two main layers: object detection and classification, which is the support of the previous one and transfer learning.

**Figure 2:**
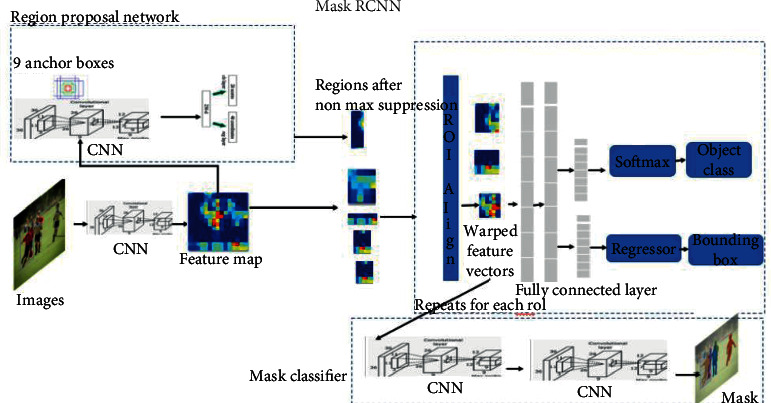
General Mask RCNN architecture.

The same Matterport Mask RCNN employed in our previous study was employed [9] in the object detection (Figure 2).

**Figure 3:**
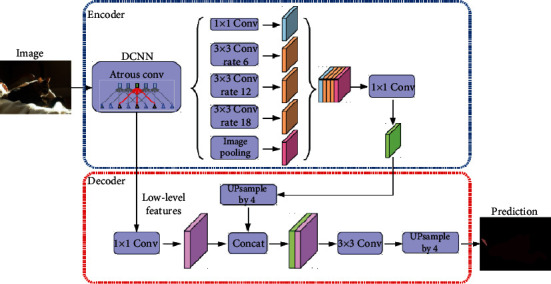
General ResNet Atrous architecture.

A ResNet101 was employed in the classification layer (Figure 3). The classification layer was the same as the previous study [9] although to improve the automatic teeth numbering a new classification level was included (COCO).

To take advantage of the precision in the location obtained in our previous study of tooth detection, it was decided to use transfer learning for this model and thus take advantage of all hyperparameters obtained. This contributed not only to a shorter training time but also to greater precision.

## 3. Results

### 3.1. Training Process

The goal of this study was to see the feasibility of correctly recognizing 32 different FDIs. Therefore, there were 33 classes (32 + background). However, employing the symmetries of the teeth in the quadrants, it was decided to work with 8 class + backgrounds, and later, a postprocess was added.

To train this neural network, 53 workouts were carried out with a minimum of 60 epochs and a maximum of 300 epochs. The duration of each execution was between 3 and 7 hours, depending on the epochs and the learning rates used in each one of them.

For each training/validation group, the learning rate and the number of epochs were varied. The number of epochs in each group varied between 4 and 20, and the learning rate was between 0.012 and 0.0014286. Depending on the chosen combination, and especially on the strategy applied in the selection of the validation group, it was possible to observe how many epochs to use.

### 3.2. Tooth Numbering Results

The neural model achieved a total loss of 6.17% (accuracy of 93.83%). This result was obtained with the parameters detailed in Table 2.

**Figure 4:**
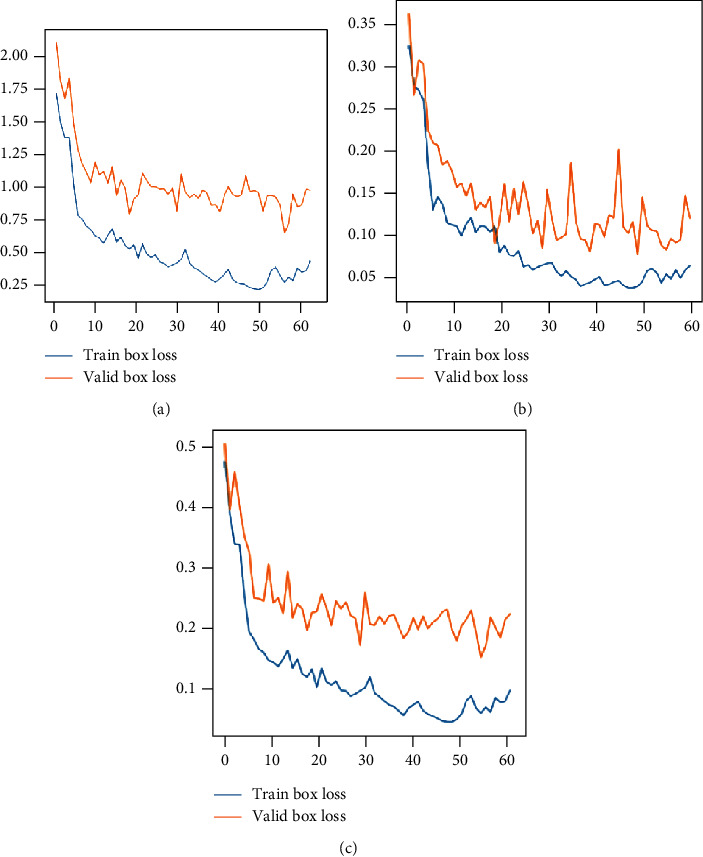
Metrics evolution: (a) total loss, (b) class, and (c) box loss of the model.

The evolution graphs of the selected metrics, both training set and the validation set, are shown in Figure 4. As can be seen in the deviation of the validation curves over the training ones in Figure 4, there is no overtraining. The blue line represents the training data's behavior, and the orange line represents the validation data's behavior.

### 3.3. Some Tooth Number Examples without Anomalies

**Figure 5:**
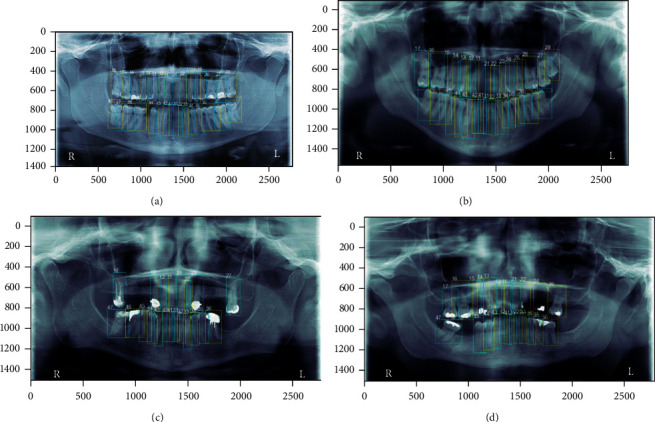
Tooth numbering: (a) image with all teeth; (b) image without teeth 46 and 36; (c) image with 21 teeth and some metallic parts; (d) image with 23 teeth and some metallic parts.

Figure 5 shows the results of tooth numbering of two images without anomalies.  Figure 5(a) shows a panoramic image with all teeth and without anomalies with a correct automatic numbering of each of the teeth.  Figure 5(b) shows a panoramic image without anomalies but with the absence of two teeth with a correct automatic numbering of each of the teeth.

### 3.4. Some Tooth Number Examples with Anomalies

**Figure 6:**
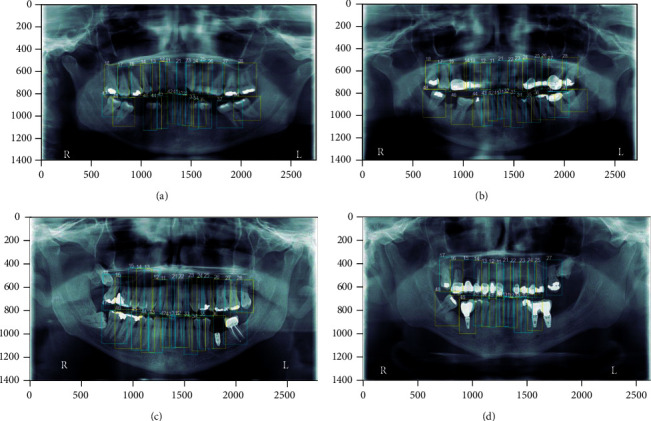
Tooth numbering: (a) image with 28 teeth with only one of the two teeth absence detected; (b) image with an absence tooth detected although the tooth exists; (c) image with two wisdom teeth not detected; (d) image with error tooth detection and pontic detected as one piece.

Figure 6 details some examples of the results provided by the neural network with some anomalies.  Figure 6(a) is an image with 28 teeth with teeth absence detected. In this case, the absence corresponds to tooth number 36 and 46, and the absence of piece 36 is detected, but tooth 46 is not detected, and tooth 46 is numbered as 47.  Figure 6(b) shows an example where the absence of 47 is detected, but nevertheless, the part exists.  Figure 6(c) is an example in which wisdom teeth are not identified in the 1^st^ and 4^th^ quadrants. In Figure 6(d), tooth number 28 is not detected, and the pontic is considered as one piece.

## 4. Discussion

This study is aimed at building a convolutional neural network to number teeth using panoramic radiographs. A Matterport Mask RCNN, ResNet101, and a transfer learning from this model were employed to achieve the objective of having the best possible accuracy. The architecture of the model achieved an accuracy of 99.24% in tooth detection and 93.83% in numbering teeth.

The neural network employed in this study was first constructed to automatically detect the presence or absence of a tooth with an accuracy of 99.24%, according to a previous author's manuscript [9]. Therefore, it was modified to add a new task which is tooth numbering employing FDI classification.

Convolutional networks have extensively been applied with very good results in image recognition tasks in several fields as medical image analysis [11], mainly in tooth detection and numbering in dental radiographs.

Several published studies have analyzed dental images with image-processing algorithms to reach high accuracy in tooth classification. These algorithms employed to classify teeth are Fourier descriptors [12], textures [13], Bayesian techniques [12], among others.

Hosntalab et al. [4] employed multistage technique to classify teeth in multislice CT (MSCT) images. The algorithm employed had three stages: segmentation, feature, and tooth classification performed by a conventional supervised classifier. A difference with the architecture proposed in this study is that this study has two layers and a transfer learning. The main advantage between both studies is that the classification result in our architecture does not rely on the accuracy of hand-crafted feature extraction algorithms.

Bitewing images are commonly used to number a tooth employing artificial intelligence [5, 14]. Chen et al. [5] employed a faster R-CNN to number teeth in periapical images. The image database in this case was 1,250 images, and teeth were numbered following the FDI system. The precision of the neural network in detecting the tooth was 98.8%, but the precision in numbering the tooth boils down to 71.5%. As in our study, the precision in tooth detection is higher than in their numbering. However, our proposed network achieves greater precision in both tasks than the one proposed by Chen et al.

Yasa et al. [14] analyzed 1,125 bitewing images with a faster R-CNN with the goal of identifying and number teeth. The proposed neural network achieved a precision of 0.9293 in tooth numbering.

Tuzoff et al. [15] employed 1,574 anonymized panoramic radiographs to detect and number teeth according to the FDI notation with a faster R-CNN algorithm. The precision in this case was 99.41% in tooth detection, and a specificity is 0.9994 in teeth numbering.

Yuniarti et al. [16] used 16 images (6 bitewing and 10 panoramic) to detect and number teeth with a method that achieved an accuracy of 91.6% in detection and 81.5% in numbering.

Sathya and Neelaveni [17] identify and number teeth in radiographic images with a transfer learning approach using AlexNet with TL. This study achieved an accuracy on molar teeth of 94.16% and 94.06%, in premolars of 93.75% and 94.25%, in canines of 86.5% and 87%, and in incisors of 91.5% and 89.5% in maxilla and mandible, respectively.

Estai et al. [18] classify permanent teeth on 591 orthopantomogram images employing CNNs and achieved a precision of 0.99.

Bilgir et al. [19] developed a Faster R-CNN to automatically number teeth on a database of 2,482 panoramic radiographs. This study achieved a precision of 0.9652.

Orhan et al. [20] employed cone-beam computed tomography (CBCT) images to detect periapical pathosis.

The mains strengths of this study are the number of images analyzed, with a total of 5,121 X-rays, which were categorized by two experts with more than three years of experience in general practice. In this sense, it is important to take into account the concordance between examiners, detailed in the previously published manuscript [9]. In addition, our neural network was trained the model with natural roots, dental implants, filled teeth, endodontic treatments, among others, so most of the clinical situations are included.

Image database contains 8,000 panoramic images with a great variety of health conditions. However, some anomalies have been obtained. For example, some of the images showed the absence of several teeth, and the network correctly identified that those teeth were missing and obtained the correct numbering. However, in other cases, the network detected the absence of a tooth, but the numbering proposal was wrong. On the other hand, the network is capable of correctly numbering teeth that contain metal parts, or any other treatment performed on it such as filled teeth, but in the case of the prosthetic crown, it detects a single tooth. This is due to how the examiners selected these types of situations.

## 5. Conclusions

Based on the final accuracy achieved both in detecting and numbering teeth, it is possible to conclude that the convolutional neural network proposed can be used in real clinical practice. The architecture of the model achieved an accuracy of 99.24% in tooth detection and 93.83% in numbering teeth.

## Figures and Tables

**Table 1:** FDI distribution in the total image database.

Q1		Q2		Q3		Q4		Total
FDI	Count	FDI	Count	FDI	Count	FDI	Count	
11	1992	21	1990	31	1996	41	1996	7974
12	1959	22	1963	32	1999	42	1999	7920
13	1956	23	1956	33	2011	43	2011	7934
14	1863	24	1859	34	1959	44	1959	7640
15	1838	25	1828	35	1921	45	1921	7508
16	1778	26	1768	36	1661	46	1661	6868
17	1793	27	1765	37	1741	47	1741	7040
18	947	28	979	38	1015	48	1015	3956

**Table 2:** Final parameters of the model.

Matterport configuration class	
Name	CoreDXnet II
Backbone	Resnet101
Batch size	2
Detection min confidence	0.75
Learning momentum	0.9
Steps per epoch	200

## Data Availability

The image data used to support the findings of this study have not been made available because of patient privacy.
